# Phenotypic Diversity and Association Mapping of Ascorbic Acid Content in Spinach

**DOI:** 10.3389/fgene.2021.752313

**Published:** 2022-01-03

**Authors:** Dario Rueda, Henry O. Awika, Renesh Bedre, Devi R. Kandel, Kranthi K. Mandadi, Kevin Crosby, Carlos A. Avila

**Affiliations:** ^1^ Department of Horticultural Sciences, Texas A&M University, College Station, TX, United States; ^2^ Texas A&M AgriLife Research and Extension Center, Weslaco, TX, United States; ^3^ Department of Plant Pathology and Microbiology, College Station, TX, United States

**Keywords:** ascorbic acid, genome-wide association, SNP markers, spinach, vitamin C

## Abstract

Ascorbic acid (AsA), or vitamin C, is an essential nutrient for humans. In plants, AsA functions as an antioxidant during normal metabolism or in response to stress. Spinach is a highly nutritious green leafy vegetable that is consumed fresh, cooked or as a part of other dishes. One current goal in spinach breeding programs is to enhance quality and nutritional content. However, little is known about the diversity of nutritional content present in spinach germplasm, especially for AsA content. In this study, a worldwide panel of 352 accessions was screened for AsA content showing that variability in spinach germplasm is high and could be utilized for cultivar improvement. In addition, a genome-wide association study for marker-trait association was performed using three models, and associated markers were searched in the genome for functional annotation analysis. The generalized linear model (GLM), the compressed mixed linear model (CMLM) based on population parameters previously determined (P3D) and the perMarker model together identified a total of 490 significant markers distributed across all six spinach chromosomes indicating the complex inheritance of the trait. The different association models identified unique and overlapping marker sets, where 27 markers were identified by all three models. Identified high AsA content accessions can be used as parental lines for trait introgression and to create segregating populations for further genetic analysis. Bioinformatic analysis indicated that identified markers can differentiate between high and low AsA content accessions and that, upon validation, these markers should be useful for breeding programs.

## Introduction

Vegetable consumption has significantly increased over the last 20 years (FAOSTAT, 2021), due in part to rising consumer interest in their nutritional properties (Wagner et al., 2016; Blekkenhorst et al., 2018). Spinach (*Spinacia oleracea* L.), of the Chenopodiaceae family, is an economically important, green, leafy crop grown around the world (Schreinemachers et al., 2018). Generally, improving nutritional content has not been prioritized in commercial spinach varieties; the predominant focus has been breeding for yield and disease resistance (Kunicki et al., 2010; Villarroel-Zeballos et al., 2012; She et al., 2018). Therefore, one current goal in spinach breeding programs is to increase crop productivity by improving disease resistance and environmental stress tolerance while enhancing quality and nutritional content.

Spinach is an annual, diploid plant (2n = 2x = 12) with an estimated genome size of 989 Mb (Arumuganathan and Earle, 1991). It is thought to have originated in Asia, specifically in Persia (Iran). Although it is clear that spinach disseminated towards China and Europe, the specific routes for this migration are still unclear (Morelock and Correll, 2008; Ribera et al., 2020). In the United States, spinach was first introduced in the 11th century by European colonists (Klockow and Keener, 2009; Correll et al., 2011; Kandel et al., 2019; Ribera et al., 2020). As a leafy green, it is consumed fresh (Klockow and Keener, 2009), cooked, or as part of other dishes (Morelock and Correll, 2008; Kandel et al., 2019). Spinach consumption can be beneficial to humans due to its high nutritional content. For example, spinach is high in vitamins A and C, as well as in several minerals including calcium, iron, and sodium (Younis et al., 2015). Spinach also contains high levels of flavonoids and the carotenoids lutein and zeaxanthin (Ribera et al., 2020). Optimizing nutrient concentrations together with consumer-preferred texture, color, and taste will further promote spinach and leafy green consumption.

Vitamin C (ascorbic acid, AsA) is an essential nutrient for humans that is involved in growth, development, and repair of tissues (Padayatty and Levine, 2016). Ascorbic acid is important for humans, as it can prevent scurvy and work as an enzyme cofactor for metabolite catabolism (Fenech et al., 2019). In plants, ascorbic acid functions as an antioxidant (Sarker and Oba, 2020a; Sarker et al., 2020b) that eliminates cell-damaging free radicals produced during normal metabolism (Sarker and Oba, 2018b; Sarker and Oba, 2018c) or in response to stress (Sarker and Oba, 2019d; Paciolla et al., 2019; Sarker and Oba, 2020f). Previous reports have shown that leafy vegetable species, like *A. hypochondriacus* (Sarker and Oba, 2020c), *A. blitum* (Sarker and Oba, 2020b), green morph amaranth (Sarker et al., 2020a) and red morph amaranth (Sarker and Oba, 2019c) are the essential sources of ascorbic acid. Furthermore, abiotic stresses, such as drought (Sarker and Oba, 2018d; Sarker and Oba, 2018e) and salinity stress (Sarker et al., 2018a; Sarker and Oba, 2018a) augmented the leaf ascorbic acid concentration of vegetable amaranth. In addition, ascorbic acid is a cofactor in plant signaling pathways related to flowering, detecting and responding to pathogen activity, and modulating the expression of metabolic genes (Ishikawa et al., 2018).

Genetic engineering efforts targeting different enzymes in the ascorbic acid pathway have resulted in enhanced ascorbic acid content. For example, overexpression of *dehydroascorbate reductase* (*DHAR*) in tobacco and maize showed that AsA content can be increased by recycling ascorbic acid from its oxidized state (dehydroascorbate) to the ROS scavenging active state (l-ascorbate) (Chen et al., 2003). Likewise, overexpressing different enzymes in the AsA synthesis pathway such as *myo*-*inositol oxidase* (*MIOX*) in Arabidopsis (Lorence et al., 2004) and *GalUR* ( *d* *-Galacturonic acid*) in potato (Upadhyaya et al., 2011) also resulted in enhanced AsA content. The ascorbic acid enriched transgenic potato plants were shown to have better tolerance to high soil salinity (Upadhyaya et al., 2011). Furthermore, the *GDP-* *d* *-mannose pyrophosphorylase* gene from tomato was inserted in tobacco plants resulting in 2–4 times higher ascorbate content as compared to wild type plants. Plants with higher AsA content showed reduced accumulation of reactive oxygen species and higher photosynthesis rates when plants were exposed to both low and high temperatures (Wang et al., 2011).

Alternatively, researchers have aimed to improve AsA content in several crops by conventional approaches (Hemavathi et al., 2009; Zhu et al., 2014; Bao et al., 2016; Kim and Lee, 2016; Abdelgawad et al., 2019; Kang et al., 2020). To facilitate molecular breeding, several groups have used genome-wide association studies (GWAS) to explore germplasm variation and to identify associations between genetic variation and phenotypic data (Tam et al., 2019). For example, a study performed in apple (*Malus* x *domestica*) revealed significant variations in AsA content found in fruit, although no significant genetic markers were associated with AsA (Lemmens et al., 2020). By contrast, a GWAS revealed 22 significant markers associated with AsA in tomato fruits across 174 tomato accessions (Zhang et al., 2016). Previous attempts to enhance AsA in model plants and several crops such as leafy vegetable amaranth, tomato, potato, and rice indicate that increased AsA content results in higher biomass and greater tolerance to abiotic stresses (Lisko et al., 2013b, Sarker and Oba, 2018e; Sarker et al., 2018a). Therefore, it is possible to simultaneously improve nutritional content, yield, and stress tolerance by enhancing AsA content in spinach.

Taking advantage of the great diversity present in spinach (Chitwood et al., 2016; Qin et al., 2017; Awika et al., 2019; Awika et al., 2020), this study aimed to identify spinach germplasm with high vitamin C content for cultivar improvement. Furthermore, since differential AsA content between accessions can be associated with genetic variation in spinach germplasm, genome-wide association analysis was performed to identify associated markers for their utilization in molecular breeding programs.

## Materials and Methods

### Plant Material

A total of 352 spinach accessions were obtained from the USDA-National Plant Germplasm System (NPGS). The USDA-NPGS accessions were previously grouped by Awika and co-authors (2019) into regions depending on their reported origin. Germplasm included 80 accessions from Turkey; 36 from the USA; 21 from Afghanistan; 18 from Macedonia; 16 from China; 15 from Iran; 11 from India; 9 from Belgium; 6 from Syria; 5 each from Hungary and Japan; 4 from France; 3 from the Netherlands, Georgia and Spain; 2 from Egypt, Ethiopia, Greece, Italy, Pakistan, South Korea, and the United Kingdom; 1 from Denmark, Germany, Iraq, Mongolia, Nepal, Poland, Serbia, the Soviet Union, Sweden and Taiwan; and 101 accessions of unknown origin.

### Growth Conditions and Experimental Design

The 352 spinach accessions were grown under controlled conditions in growth chambers at the Texas A&M AgriLife Research and Extension Center located in Weslaco, Texas. The growth chambers were set to an average temperature of 23°C, 11 h of light, and a light intensity of 120 µmol/m^2^/s.

The spinach lines were sown in 500 cc pots that contained BM2 germination mix (Berger, Saint-Modeste, QC) supplemented with Osmocote classic 14-14-14 slow-release fertilizer (ICL specialty fertilizers, Dublin, OH) in a proportion of 82:1 v/v. Spinach plants were grown for 2 months with daily watering as needed and fertilized weekly with Water Soluble Tomato Plant Food (Miracle-Gro, Marysville, OH). Accessions were divided across 24 different trays. Each tray contained three pots of spinach cv Viroflay as a control for normalizing data and 15 different test accessions, for a total of 18 pots per tray. Three biological replicate plants per accession were evaluated.

### Tissue Collection

Leaf tissue (150 mg) from 2-month-old plants was collected to measure ascorbic acid content. Tissue collection was performed before noon. Collected leaf tissue was placed in 15 ml centrifuge tubes, flash frozen in liquid nitrogen, and stored at −80°C until AsA quantification.

### Quantification of AsA Content

AsA content was quantified using a previously described ascorbate oxidase method (Lorence et al., 2004). Briefly, two technical replicates were performed. Tissue was homogenized in a MiniG™ 1,600 grinder (Spex^®^SamplePrep, Metuchen, NJ) for 35 s at 1,500 rpm, using a pre-cooled aluminum block to avoid tissue thaw. Six premium grade BBs (Daisy^®^, Rogers, AR) were added to each tube to aid grinding. Immediately after grinding, 2.25 ml of ice-cold 6% metaphosphoric acid was added to each tube, followed by mixing. Then, 0.75 ml of the plant extract was transferred to 1.5 ml conical tubes in duplicate to create the technical replicates. Tubes were centrifuged at 16 g for 5 min at 4°C, and the supernatant was decanted into new 1.5 ml centrifuge tubes. During the whole process, samples were kept on ice and away from direct light to prevent ascorbic acid oxidation.

Oxidized and reduced AsA contents in the samples were measured spectrophotometrically at 265 nm absorbance using 10 mm pathlength UV cuvettes (Brand GMBH + CO KG, Wertheim, Germany) and a NanoDrop OneC spectrophotometer (Thermo Fisher Scientific, Waltham, MA). The spectrophotometer was blanked with 1 ml of 0.1 M K-phosphate buffer in disposable UV cuvettes. Samples were prepared by adding 950 µl of K-phosphate buffer +50 µl of plant extract into new UV cuvettes and initial absorbance was recorded. To quantify oxidized AsA, 1 µl of 1 mM dithiothreitol (DTT) was added and samples were mixed. The UV cuvettes were placed in a dark environment and incubated for 20 min at room temperature before recording the final absorbance. Reduced ascorbate was quantified similarly, but instead of DTT, 1 unit/µl ascorbate oxidase was used.

The total ascorbate content was calculated with the previously reported formula (Lorence et al., 2004):

$$\begin{array}{l} \text{Total}\;\text{change}\;\text{in}\;\text{absorbance}\;=\;\left[\text{Reduced}\;\text{ascorbate}\right]\;+\;\left[\text{Oxidized}\;\text{ascorbate}\right]\\ \text{Total}\;\text{ascorbate}\;\text{content}=\left\{\frac{\left[\frac{\left(\text{Total}\;\text{change}\;\text{in}\;\text{absorbance}\right)}{14.3}\right]\times \;20\;\times \;0.75}{\left(\text{Sample}\;\text{weight}\;\text{in}\;\text{grams}\right)}\right\} \end{array}$$

### Single Nucleotide Polymorphism Markers

SNPs reported by (Awika et al., 2019) were utilized for genome-wide association mapping. SNP markers were obtained by a ddRADseq genotyping-by-sequencing (GBS) protocol. A total of 6,167 SNPs were utilized, with the highest number (1,458 SNPs) located at chromosome 4 (Chr4) followed by Chr3, Chr1, and Chr2 (1,060, 1,027, and 1,027 SNPs, respectively). Chromosome 6 and 5 had the fewest SNPs, with 838 and 757 SNPs, respectively (Awika et al., 2019).

### Population Stratification and Kinship

Allele frequency differences can occur between subpopulations and such population stratification, if not accounted for, can lead to false positive associations and/or mask true associations (Falush et al., 2007; Alexander et al., 2009). Population structure (stratification) was estimated using the allelic ancestry-based admixture model (Falush et al., 2007; Alexander et al., 2009) in STRUCTURE Version 2.3.4 (Pritchard et al., 2000). The parameters used in STRUCTURE were 10,000 burn-ins with 1,000 replications on the 6,167 SNPs and 15 runs for the K values from 1 to 10. The optimal value of K (i.e., the number of computation clusters) of the inferred population structure (Q) was established using the Evanno ΔK method (Evanno et al., 2005). Briefly, the optimal cluster was identified by the highest value at the change of K (Δ*K*). The genetic variance and error variance were determined for each compression level (likelihood), and the one with the lowest −2LnLk for the trait–compression combination value was used to test the markers. A kinship matrix (k) was created from the SNP data to account for hidden relatedness among individuals by allele sharing as described Blouin (2003), (Endelman and Jannink, 2012). Such a correction is important to reduce biased estimations of standard errors of SNP effect sizes. Estimation of kinship was calculated using the default method, Centered-IBS (identity by state) and Max 6 Allele Frequency using the ‘Kinship’ plugin in TASSEL 5.2.54 (Bradbury et al., 2007).

### Genome-wide Association Methods

The best linear unbiased prediction values (BLUPs) values were estimated using the software JMP^®^ version 14.0 (JMP^®^, 2019). Three linear models were used in the association study: 1) the generalized linear model (GLM); 2) the compressed mixed linear model (CMLM, Q + *k*) on a per trait basis to estimates genetic and residual variance at the trait level; and 3) the CMLM on a per marker trait basis, for estimates of genetic and residual variance at the marker level (Awika et al., 2019). Association models were run in TASSEL v5.2.54 (Bradbury et al., 2007). For GLM, max *p* value 1.0 and minimum class size 0 were set. In both CMLMs, the Q was treated as covariate and k as a random coefficient (Kumar et al., 2018). Compression in CMLM reduces computing time and increased statistical power, while P3D does not affect statistical power but decreases computing time (Zhang et al., 2010). For CMLM association analysis, the optimum level of compression was set as Population Parameters Previously Determined (P3D) or Re-estimate after each marker (per Marker). To account for the false discovery rate, thresholds for markers significantly associated with AsA content in the three models were established using the Benjamini-Hochberg (Benjamini and Hochberg, 1995) multiple comparison method at α = 0.05. The calculated thresholds were 4.44E−04, 3.37E−04, and 2.56E−04 for GLM, P3D, and perMarker, respectively.

### Finding Genomic Features Anchoring Significant Markers

The genomic features anchoring near or on significant associated SNPs were identified using the genomic browser www.spinachbase.org (Collins et al., 2019), which contains the partially sequenced spinach genome. Genomic features were tabulated individually in the browser by identifying the gene nearest to the position of the SNPs.

### Functional Enrichment Analysis of Anchored Genes and Phylogenetic Analysis of High- and Low- AsA Genotypes

Functional enrichment analysis of the input spinach genes was performed based on the gene ontology (GO) terms and gene families represented. The agriGO (Du et al., 2010) and GenFam (Bedre and Mandadi, 2019) web tools were used to analyze statistically enriched GO terms (biological process, molecular functions, and cellular components) and gene family categories using the Fisher exact test. The Benjamini-Hochberg (Benjamini and Hochberg, 1995) approach was used to adjust the *p* values for multiple testing in the GO and GenFam analyses. Phylogenetic analysis of genotypes was performed using TASSEL v5.2.54 (Bradbury et al., 2007). A phylogenetic tree was constructed using MEGA X (Kumar et al., 2018) utilizing neighbor-joining statistical method and bootstrap method with 500 bootstrap replications as a test of phylogeny.

## Results and Discussion

### High Variation of Leaf Ascorbic Acid Content in Germplasm Indicates Good Potential for Improvement via Breeding

**FIGURE 1:**
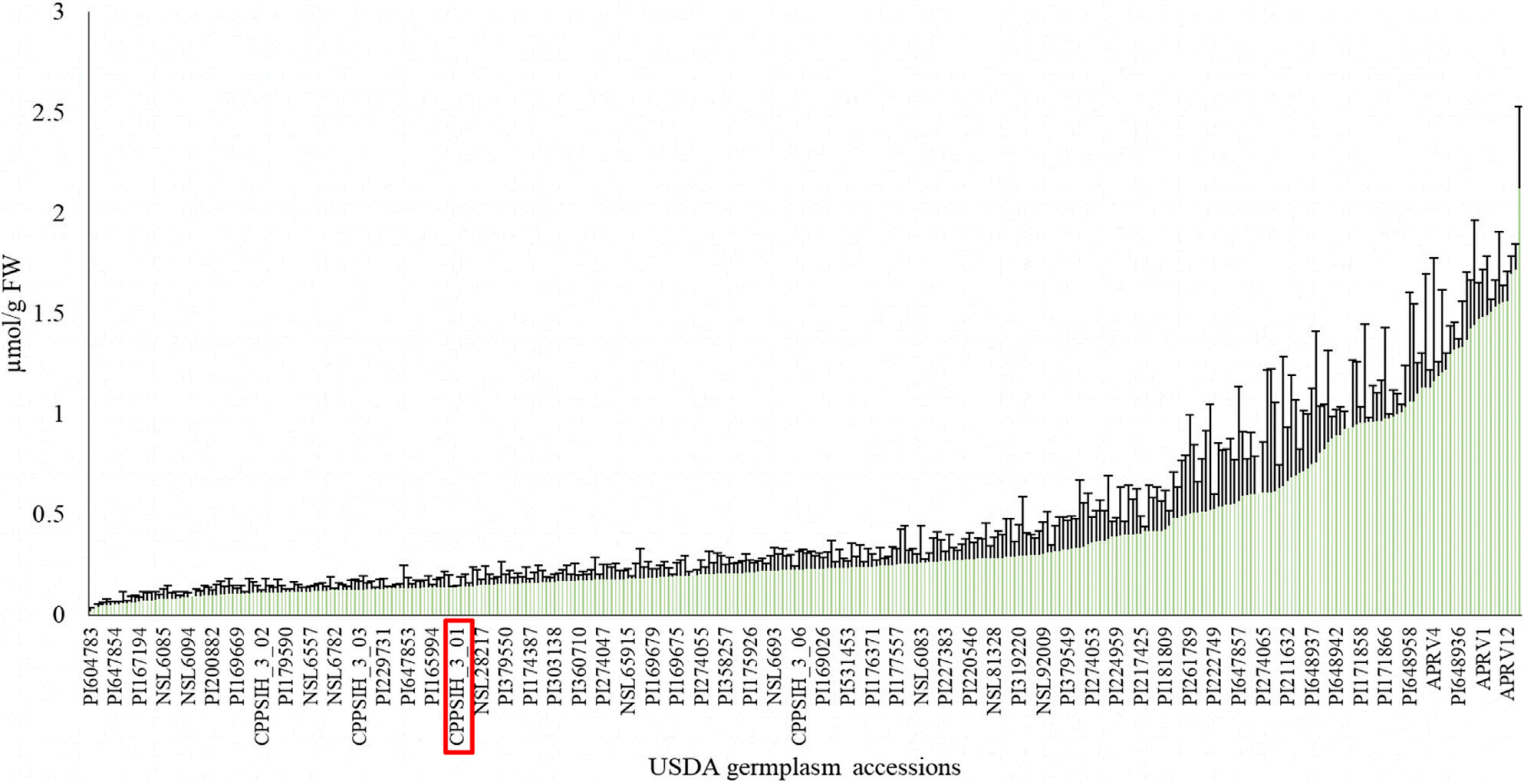
Phenotypic diversity of ascorbic acid content in USDA-NPGS spinach germplasm. The red box indicates the commercial cv Viroflay. Due to space limitations, only a few accession IDs are labelled (Mean value + SD, *N* = 3).

This study evaluated AsA content in a collection of 352 spinach accessions obtained from the USDA-NPGS representing a cross-section of spinach genetic diversity. For each accession, three biological replicates (3 pooled plants per replicate) and two technical replicates per biological replicate were measured. The mean of the population’s AsA content was 0.3766 µmol/g fresh weight (FW), with a range of 0.02–2.12 µmol/g FW (Figure 1, Supplementary Table S1). The average coefficient of variation within accessions in the population was 61.58%, indicating high AsA content variability not only between accessions but also within plants in the same accession, most likely due to the dioecious nature of spinach. The commercial control cultivar Viroflay had an AsA content of 0.1414 µmol/g FW, a value below the population average, which suggests that there is an opportunity to enhance AsA content in commercial varieties of spinach using available genetic diversity.

Similar results have been reported in other crops where diversity of AsA content has been measured. Lisko and collaborators (2013a) evaluated 24 rice (*Oryza sativa*) accessions from US, Japan, Taiwan, and the Philippines showed a broad spectrum in AsA diversity. Foliar measurements at the vegetative 2 (V2) developmental stage ranged from 1.5-8 µmol/g FW in various accessions (Lisko et al., 2013a). Likewise, high variability was found when tomato (*Solanum lycopersicum*) germplasm was analyzed for AsA content in the fruit ranging from 0.069-2.203 µmol/g FW in round tomatoes and 0.185–1.847 µmol/g FW in cherry types (Bhandari et al., 2016).

Other leafy greens have been also screened for AsA content. A wide range of variations in ascorbic acid content were also observed in different leafy vegetable amaranth species (Sarker et al., 2014; Sarker et al., 2015a; Sarker et al., 2015b; Sarker et al., 2016) and pigmented vegetable amaranth (Sarker et al., 2014; Sarker et al., 2018c). Llorach and co-authors (2008) screened for vitamin C content in five lettuce (*Lactuca sativa* L.) varieties and escarole (*Cichorium endivia* var. *crispa*), obtaining a AsA content range from 0.1589-1.1066 µmol/g FW (Llorach et al., 2008). In another study of one cultivar each of lettuce, white cabbage (*Brassica oleracea* L. var *capitata* L.), Chinese cabbage (*B. chinensis* L.) and mugwort (*Artemisia vulgaris* Cantley), these crops had AsA measurements of 0.1418 µmol/g FW, 1.0669 µmol/g FW, 1.4358 µmol/g FW and 1.9920 µmol/g FW, respectively (Bahorun et al., 2004). Taken together, this study in spinach and other reports in leafy greens (Bahorun et al., 2004; Llorach et al., 2008), fruits (Bhandari et al., 2016), and grains (Lisko et al., 2013a) indicate there is high natural variation in AsA content between cultivars of many crops, and suggest a potential for improvement.

**FIGURE 2:**
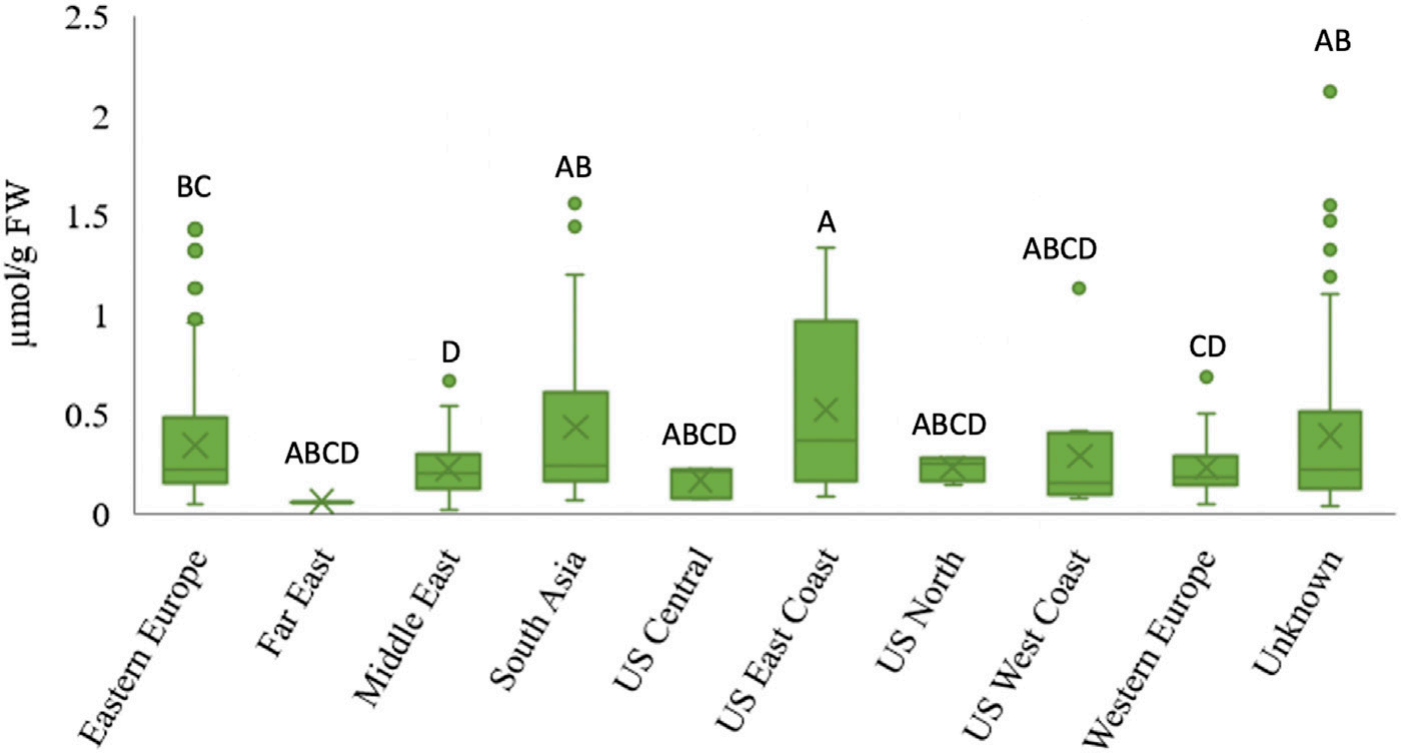
Relationship between geographical origin and ascorbic acid content in spinach germplasm. The “X” denotes the region mean, the boxes represent the inter-quantile range, the line between boxes is the median, and the dots show the outliers outside the region maximum. Different letters indicate statistical differences between regions at α = 0.05.

Data was grouped by geographic origin to determine whether there is a relationship between accession origin and AsA content (Figure 2). While statistical differences were observed (Student’s t-test, *p* < 0.05) in AsA content between regions, there were no clear, geographic trends. Similarly, no relationship between diversity in ascorbic acid content and geographical origin was observed in leafy vegetable amaranth (Sarker et al., 2017; Sarker et al., 2018b). However, it was observed that spinach accessions from Eastern Europe, South Asia, and the US East Coast had more variability in AsA content than accessions from other regions, due largely to a larger AsA content range. It has not been studied yet whether the diversity of AsA content between regions results from unintentional or targeted breeding efforts. This result is consistent with several other spinach studies that found no relationship between phenotype and geographic origin when analyzing other traits such as oxalate concentration (Shi et al., 2016b), leafminer resistance (Shi and Mou, 2016), mineral element concentration (Qin et al., 2017) leaf surface texture, petiole color and edge shape (Ma et al., 2016), and bolting, plant height, and leaf erectness (Chitwood et al., 2016).

### Population Structure Analysis Classifies 270 Spinach Accessions Into Two Population Groups

**FIGURE 3:**
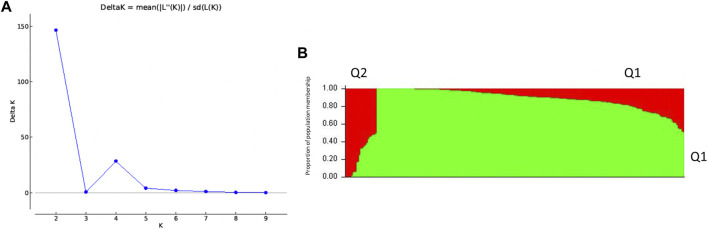
Population structure analysis classifies 270 spinach accessions into two population groups based on Δ*K* analysis **(A)** Likelihood estimate plot showing the number of genetically distinct clusters (K); **(B)** two populations (Q1 and Q2) estimated from the admixture model (individual accessions on the horizontal axis and the assigned probability on the vertical axis).

For population structure and downstream analysis, 270 out of the 352 AsA-screened accessions were selected due to availability of genotypic data. The population structure of the panel of 270 spinach accessions was estimated with the software STRUCTURE v2.3.4 for K ranging from 1 to 10. Using the highest Δ*K* value in STRUCTURE HARVESTER (Earl and Vonholdt, 2012), subpopulation K = 2 was selected (Figure 3), with K representing the number of true clusters. These results indicate that our spinach accessions are divided into two populations. A membership probability cutoff (Q value) of 0.60 was used to divide the spinach panel into two (Q1 and Q2) main populations, while the remaining accessions showing membership proportions Q < 0.60 formed an admixed group (Qm). The population Q1 consisted of 237 accessions, with the largest number (79 accessions, 33.33%) from East Europe. The population Q2 consisted of 19 accessions; nine are from South Asia (47.37%). The remaining 14 accessions fell in the admixture group Qm. A recent study also reported two main populations in spinach when using same set of genomic markers (Awika et al., 2019). Likewise, another study reported a two-population structure (Q1 and Q2) using same population of spinach accessions but a different set of SNP markers to perform an association analysis for oxalate concentration (Shi et al., 2016b). However, an association study investigating the mineral concentration in spinach accessions found the data supported four populations in their 292 accessions (Qin et al., 2017). Subpopulation clusters can therefore change depending on the subset of accessions used within a population and the SNPs used in a study.

### GWAS Models Identify Unique and Overlapping Markers Associated With Ascorbic Acid Content in Spinach

A total of 6,167 previously reported SNP genetic markers (Awika et al., 2019) were assessed to identify association with AsA content. The data were analyzed in TASSEL Version 5.2.54 (Bradbury et al., 2007) using the generalized linear model (GLM) (Nelder and Wedderburn, 1972), the compressed mixed linear model (CMLM) with the population parameters previously determined (P3D) approach (Zhang et al., 2010), and the perMarker model (Yu et al., 2006).

**FIGURE 4:**
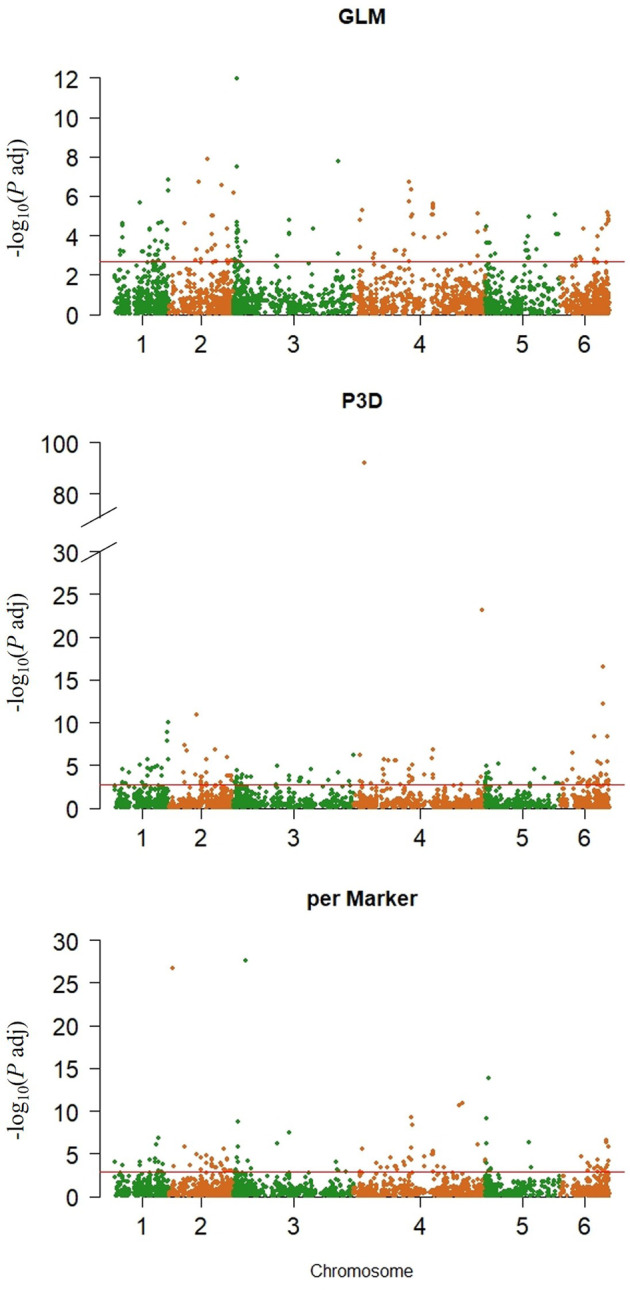
SNP markers significantly associated with ascorbic acid content in spinach. Left, Manhattan plots showing adjusted *p* value threshold and chromosome distribution by association model. Significance thresholds (adjusted *p* value) were calculated with Benjamini-Hochberg (Benjamini and Hochberg, 1995) multiple comparison method (red horizontal lines Generalized linear model (GLM), compressed mixed linear model (CMLM) with Population parameters previously determined (P3D) and CMLM with re-estimation of variance component after each marker (perMarker).

**FIGURE 5:**
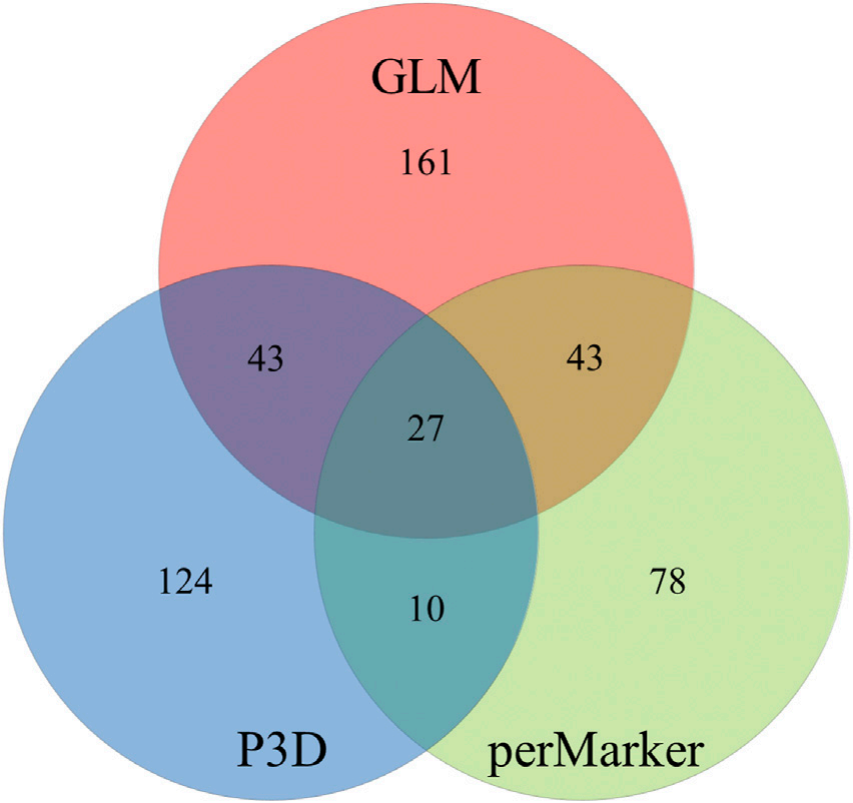
Unique and overlapping markers associated with ascorbic acid content in spinach. Generalized linear model (GLM), compressed mixed linear model (CMLM) with population parameters previously determined (P3D) and CMLM with re-estimation of variance component after each marker (perMarker).

The GLM identified 274 significant markers, the highest number of AsA-associated markers, followed by 204 markers found by the P3D, and 158 markers identified by the perMarker model (Supplementary Table S2; Figure 4). A total of 490 SNPs were identified by at least one of the three models (Figure 5); however, only 27 SNPs were commonly identified by all three models. An additional 43 SNPs were identified by both GLM and P3D, 43 SNPs were identified by GLM and perMarker, and 10 SNPs were identified by P3D and perMarker. The GLM identified the highest number of unique markers (161 SNPs), as compared with the P3D (124 SNPs) and the perMarker model (78 SNPs) (Supplementary Table S2, Figure 5).

Similar to the results obtained in this study, other groups have discussed differences in the number of associated markers identified by models, indicating advantages and disadvantages of each approach (Yu et al., 2006; Zhang et al., 2010; Wang et al., 2014). In general, false positives are often the main concern when reporting significantly associated markers in associations studies, and the GLM is known to report false positives since population structure and kinship are not simultaneously integrated during the analysis (Wang et al., 2014). However, the computing time of the GLM is reported to be very low compared to mixed linear models (MLMs) (Wang et al., 2014). Alternatively, the compressed MLM (CMLM) with re-estimation after each marker (perMarker), has been shown to reduce the computing time and to improve statistical power via compression by clustering individuals into groups. In this model, run time is proportional to the number of groups cubed, rather than the number of individuals. However, it is known that the perMarker model requires re-estimating variance components after each marker. A final approach used with the CMLM is population parameters previously determined (P3D), which improves computing time but does not affect statistical power (Zhang et al., 2010). Both forms of CMLM have an additional disadvantage when small samples are used, as they tend to produce inaccurate and irrelevant estimations of polygenic factors (Yu et al., 2006). Therefore, since each model can potentially result in false positive associations, the combination of several methods as performed in this study can help to narrow down candidates by selecting significant signals detected by more than one method.

Many association studies had been performed in spinach and other crops. Shi et al. (2016a) assessed resistance to Stemphylium leaf spot (*Stemphylium botryosum* f. sp. *spinacia*) among 273 spinach accessions. A GLM and MLM, both using Q as population structure, were performed to identify significant SNPs. The GLM identified 14 SNPs associated with pathogen resistance, but the MLM only identified seven SNPs (Shi et al., 2016a). Another association study for root morphology traits in maize also used both a GLM and MLM. A total of 297 inbred maize lines were used for this analysis and several root traits were measured. They report 355 SNPs from the GLM and 28 SNPs from the MLM (Wang et al., 2019). Ma and co-authors (Ma et al., 2016) concluded that the GLM in general could detect more markers associated with traits of interest when compared with the MLM. Results from present work in spinach support this conclusion.

Here, 27 SNPs were significantly associated with AsA levels by all three models (GLM, P3D, and perMarker) (Supplementary Table S2; Figure 5). No major QTL associated with AsA content was detected within commonly associated markers, since all 27 SNPs had low average r^2^ values for all models (8% GLM; 13% P3D; 18% perMarker). Some SNPs identified by the perMarker and P3D models had much higher r^2^ values. The r^2^ of these SNPs might be related to their observed *p*-value, because they all had the lowest *p* values among the markers identified by the respective model. One example of this was the marker 37554_100 which had a r^2^ of 87% with the model perMarker but only 8%, and 9% with GLM, P3D, respectively. Other association studies have reported low r^2^ values in situations where the trait of interest is probably controlled by a group of several minor genes (Shi et al., 2016a; Wu et al., 2020).

There may be false positive markers identified by the GLM method in this study, given that this model is known to have less control over false positives, and our GLM model reported more SNPs significantly associated with AsA than the two other models (P3D and perMarker). Within the two CMLM models, only 37 SNPs were associated with AsA content by both models (10 P3D and perMarker only, 27 shared with GLM model). Therefore, both CMLMs are likely not effectively controlling false positives and false negatives. In future work, the use of tree models might be more effective to analyze this type of data since it could control false positives and avoid declaring false negatives. Despite these caveats, the 27 AsA-associated SNPs markers identified by all three models could be primary targets for validation by researchers working to improve AsA content in spinach.

### Markers Associated With Ascorbic Acid Content Are Dispersed Throughout the Genome

**FIGURE 6:**
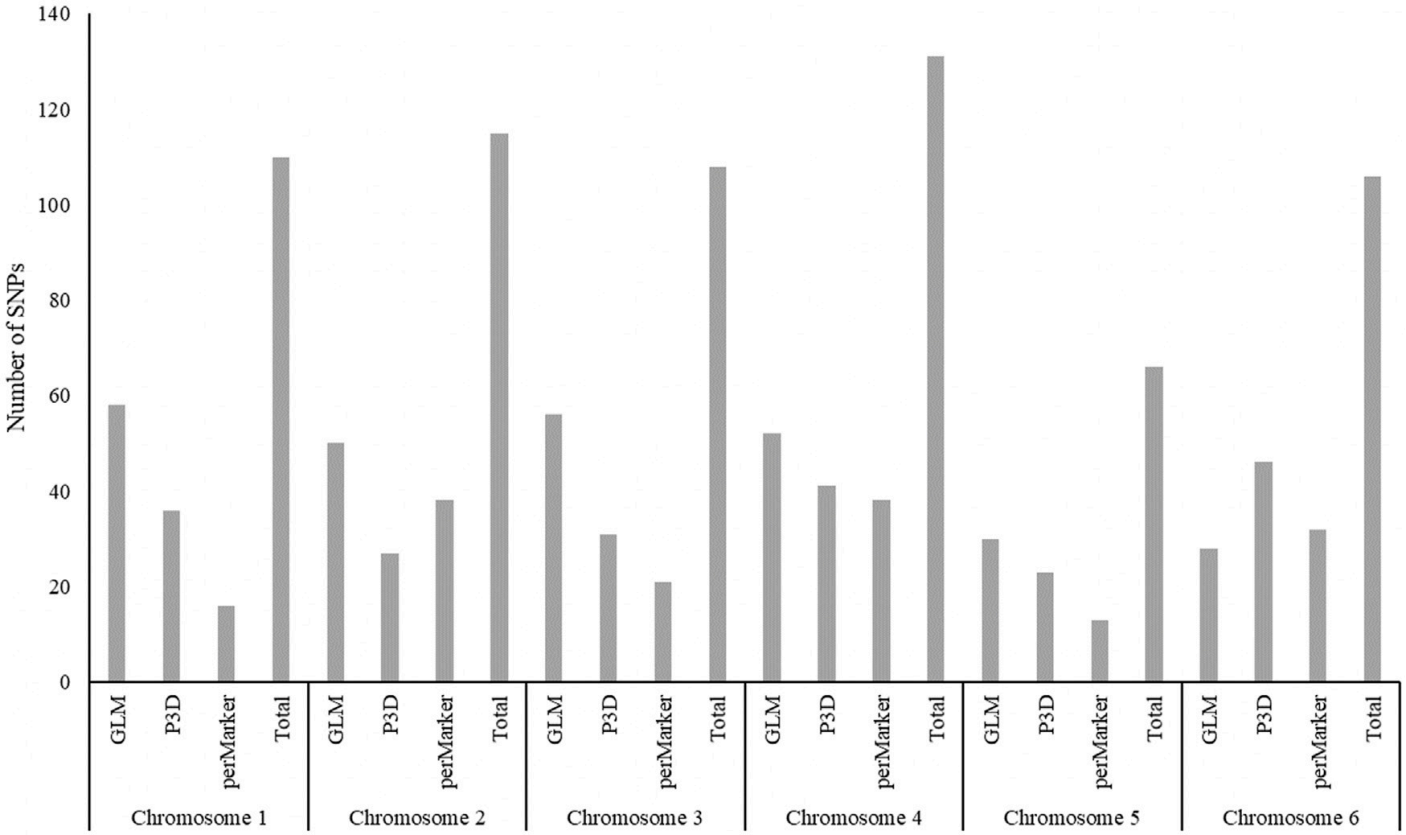
Number of ascorbic acid content–associated SNPs distributed across the spinach genome.

The 490 significant, AsA-associated SNPs we identified were distributed along the six chromosomes of the spinach genome. Of those markers, we found an almost even distribution across Chr1, Chr2, Chr3, Chr4 and Chr6–17.76, 18.57, 17.96, 18.37 and 17.35%, respectively. Chr5 had fewer markers than the other chromosomes, only 10% of identified SNPs (Figure 6). The distribution of significant SNPs across the whole spinach genome could be related with the presence of four different biosynthetic pathways for AsA (Suza et al., 2010).

When AsA quantification and a subsequent association study was performed in tomato fruits, the authors found five significant SNPs among four chromosomes (Sauvage et al., 2014). Genes that had been previously reported to be involved in ascorbic acid biosynthesis were mapped in introgression lines of tomato and were found to be spread across nine chromosomes (Zou et al., 2006). Furthermore, the *VTC* genes, which are directly involved in AsA biosynthesis, were mapped in *Arabidopsis thaliana* mutants. Four vitamin C synthesis genes (*VTC* genes), *VTC1*, *VTC2*, *VTC3*, and *VTC4*, were mapped onto Chromosome 2, 4, 2, and 3, respectively (Conklin et al., 2000). Therefore, although other studies have identified less SNPs associated with AsA, they were also found on several chromosomes in agreement with AsA content in spinach results.

### Genes Containing the Associated SNPs Show a Wide Range of Functions With Putative Roles in Biosynthesis Processes and Response to Stress

Genomic positions of the significantly associated SNPs were used to identify putative genes using the webpage www.spinachbase.org. Using the 490 significant SNPs from the three association models, 310 candidate genes were identified. Some genes had more than one significant marker. For example, three markers identified by the GLM model (*20248_143, 20250_0 and 34990_9*) were anchored within the same gene. In total, 113 unique markers that were identified by the GLM model fell within gene sequences, and an additional 51 markers were within 146 kb of a gene. When analyzing SNPs from the P3D model, 95 unique markers fell within the sequence of a gene, and 29 further unique markers were within 51.23 kb of an annotated gene. Finally, when using the perMarker method, 57 unique markers were anchored within a gene sequence, and 22 markers were within 180.62 kb of a gene (Supplementary Table S3).

Anchored markers were identified by multiple modelling methods. Out of the 43 SNPs identified by both the GLM and P3D models, 30 markers were within genes, and 13 markers were within 37.7 kb of a gene. Similarly, of the 43 SNPs identified by both the GLM and perMarker models, 33 markers were within a gene sequence, and the other 10 markers were within a distance of 191.85 kb of a gene. All 10 markers identified by both the P3D and perMarker methods were within a gene sequence. Finally, 12 of the SNPs identified by all three models were found within a gene, and the remaining 15 markers were within 191.64 kb of a gene (Supplementary Table S3).

Out of the 310 marker-anchored, reported genes, 291 genes encode known proteins (Supplementary Table S3). Notably, our analysis did not identify any genes that are directly involved in AsA biosynthetic pathways due to the random set of SNP makers available for this study. Alternatively, it is also possible that observed variability in AsA content may be due to substrate availability or plant hormone regulation rather than differences in AsA biosynthetic enzyme activity. Future work out of the scope of this project needs to test these hypotheses. However, in either case, identification of QTLs associated with high AsA is useful for molecular breeding to improve gain in selection independently of identifying the gene or set of genes directly involved in high AsA content in spinach.

Since AsA has many different functions in plants, identified genes can putatively participate in different processes and functions. AsA can work as an antioxidant in scavenging ROS (Sarker and Oba, 2020f; Sarker and Oba, 2018c), it is part of many metabolic processes (Sarker and Oba, 2018b) as an enzyme cofactor, and it is part of several signaling pathways including those that control the flowering period, responses to active pathogen activity and the expression of metabolic genes. The leaf ascorbic acid concentration of vegetable amaranth reported to be enhanced under abiotic stresses, such as drought (Sarker and Oba, 2018d) and salinity stress (Sarker et al., 2018a; Sarker and Oba, 2018a). It revealed from the literature that ascorbic acid had a strong relation to the antioxidant activity of different vegetable amaranth species, such as *A. gangeticus* (Sarker and Oba, 2020e), *A. tricolor* (Sarker and Oba, 2020d), stem amaranth (Sarker et al., 2020c), *A. lividus* (Sarker and Oba, 2019a) and *A. spinosus* (Sarker and Oba, 2019b). Therefore, the identified genes were grouped by biological process, molecular function, and putative gene family using the agriGO (Du et al., 2010) and GenFam (Bedre and Mandadi, 2019) web tools, which use the Fisher exact test to analyze enriched GO and gene family categories.

**FIGURE 7:**
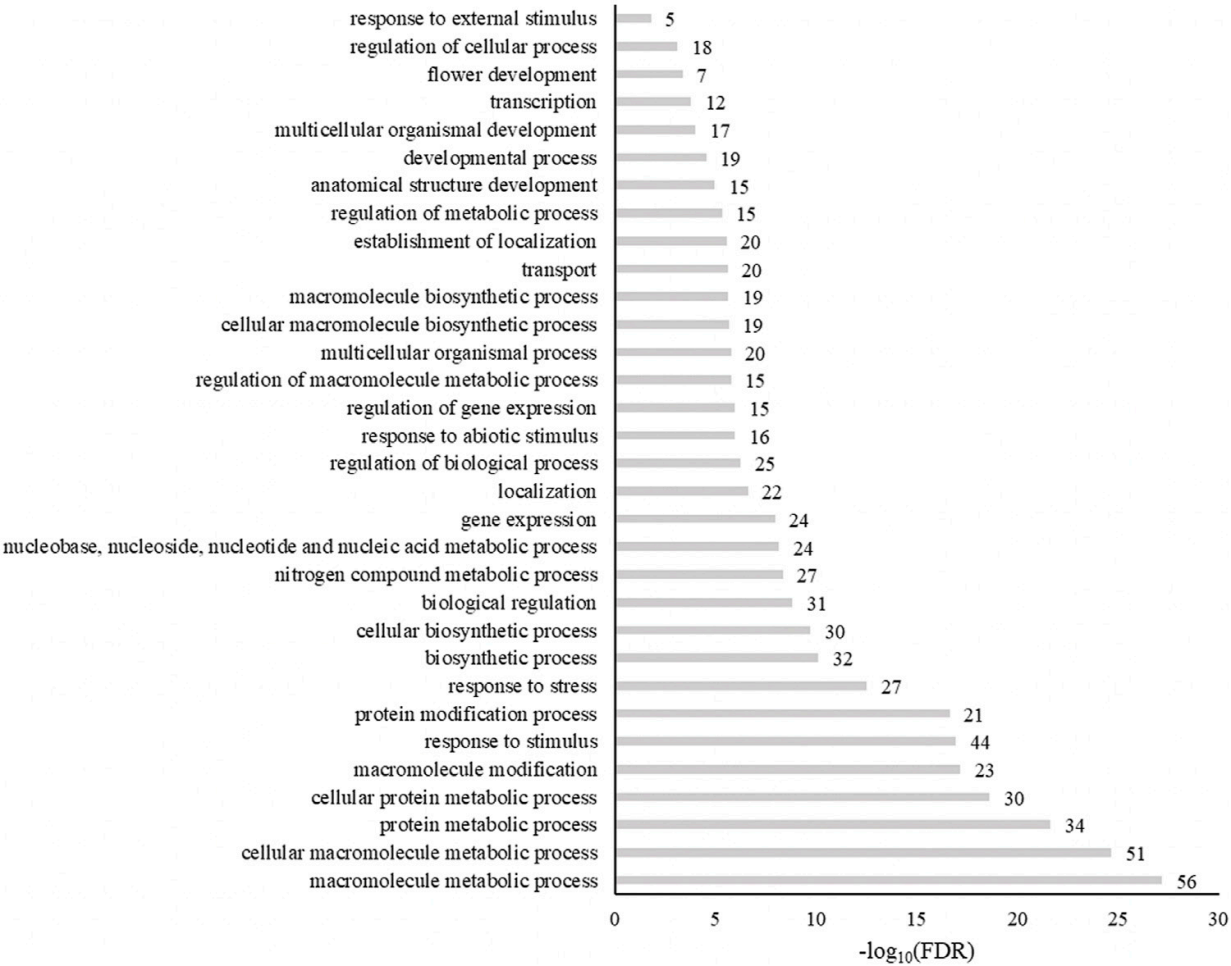
Enrichment of gene ontogeny classification of genes anchored to spinach ascorbic acid content–associated markers by biological process. Numbers next to bars are the genes related to that process.

**FIGURE 8:**
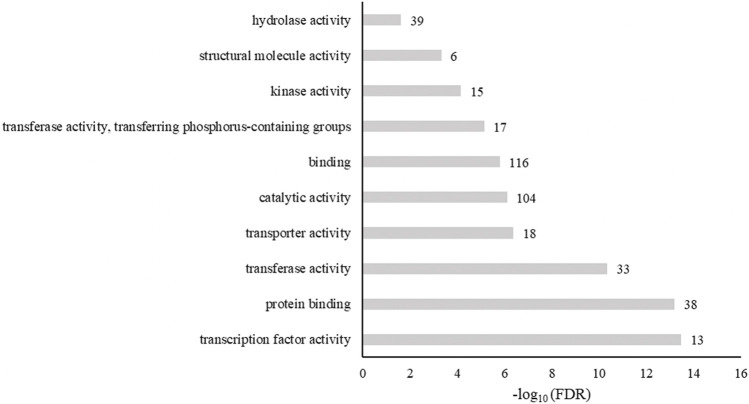
Enrichment of gene ontology classification of genes anchored to spinach ascorbic acid content–associated markers by molecular function. Numbers next to bars are the genes related to that function.

The biological processes enriched in these genes included metabolic processes, responses to a stimulus, responses to stress, biosynthetic processes, and biological regulation (Figure 7). When genes were grouped by molecular functions, SNP-anchored genes are enriched for transcription factor activity, protein binding, transferase activity, and transporter activity (Figure 8).

**FIGURE 9:**
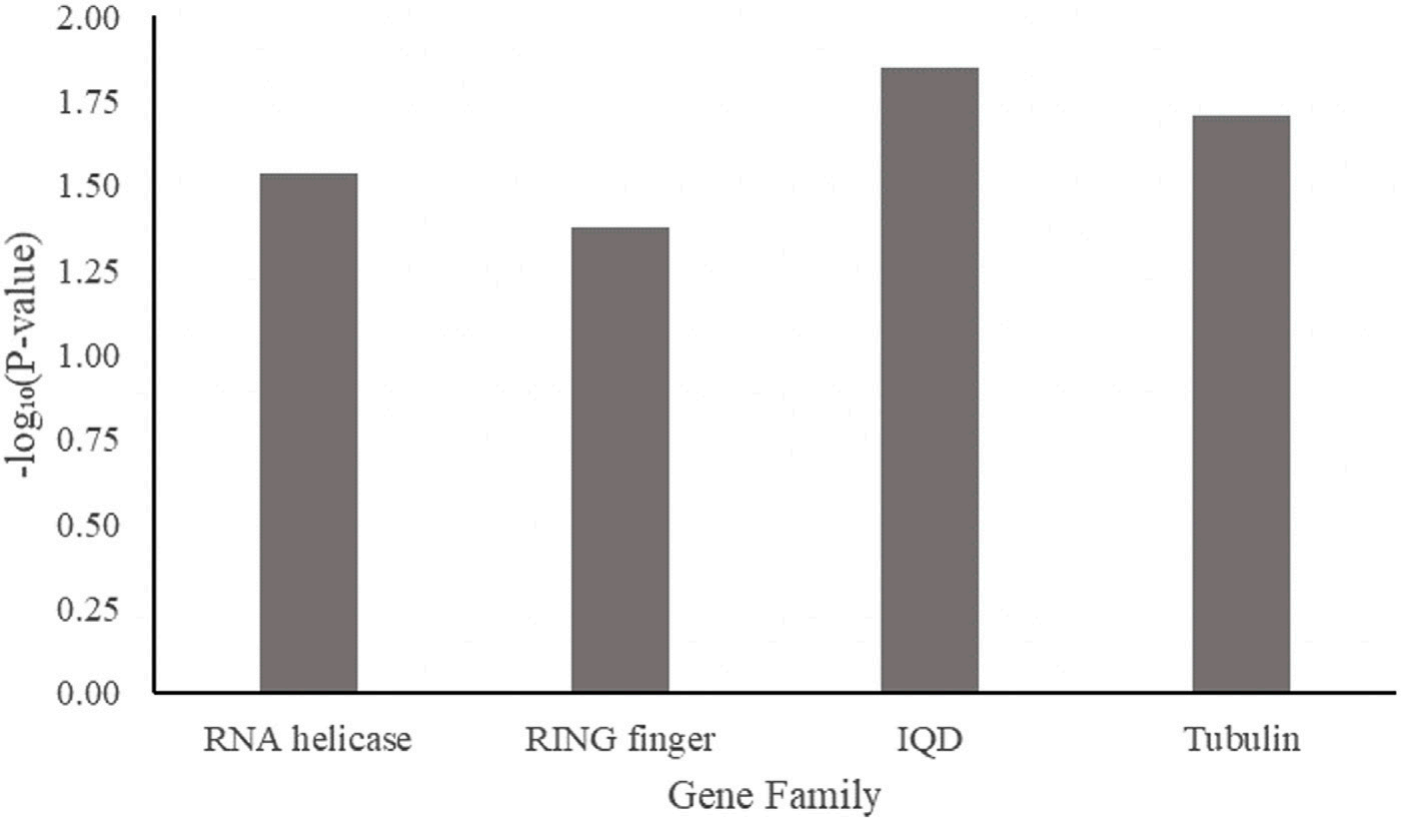
Overrepresented gene families among spinach ascorbic acid content–associated genes.

To narrow down the genes most closely related to AsA synthesis in spinach, a GenFam analysis (Bedre and Mandadi, 2019) for gene family discovery was performed. Four gene families were significant (*p* < 0.05): the RNA helicase gene family, the RING finger domain gene family, the IQD (IQ67-domain) gene family, and the tubulin gene family (Figure 9). While these gene families have been reported to be involved in AsA pathways (Wheeler et al., 1998; Dreher and Callis, 2007; Gao et al., 2011; Sharma et al., 2012; Yang et al., 2019; Zhang et al., 2020). Putative roles of these families need to be tested using functional genomic approaches to determine their involvement in AsA content regulation.

### The Relationship Between High- and Low-Ascorbic Acid Content Spinach Accessions Tested Using the 490 Significant Polymorphic Variants

**FIGURE 10:**
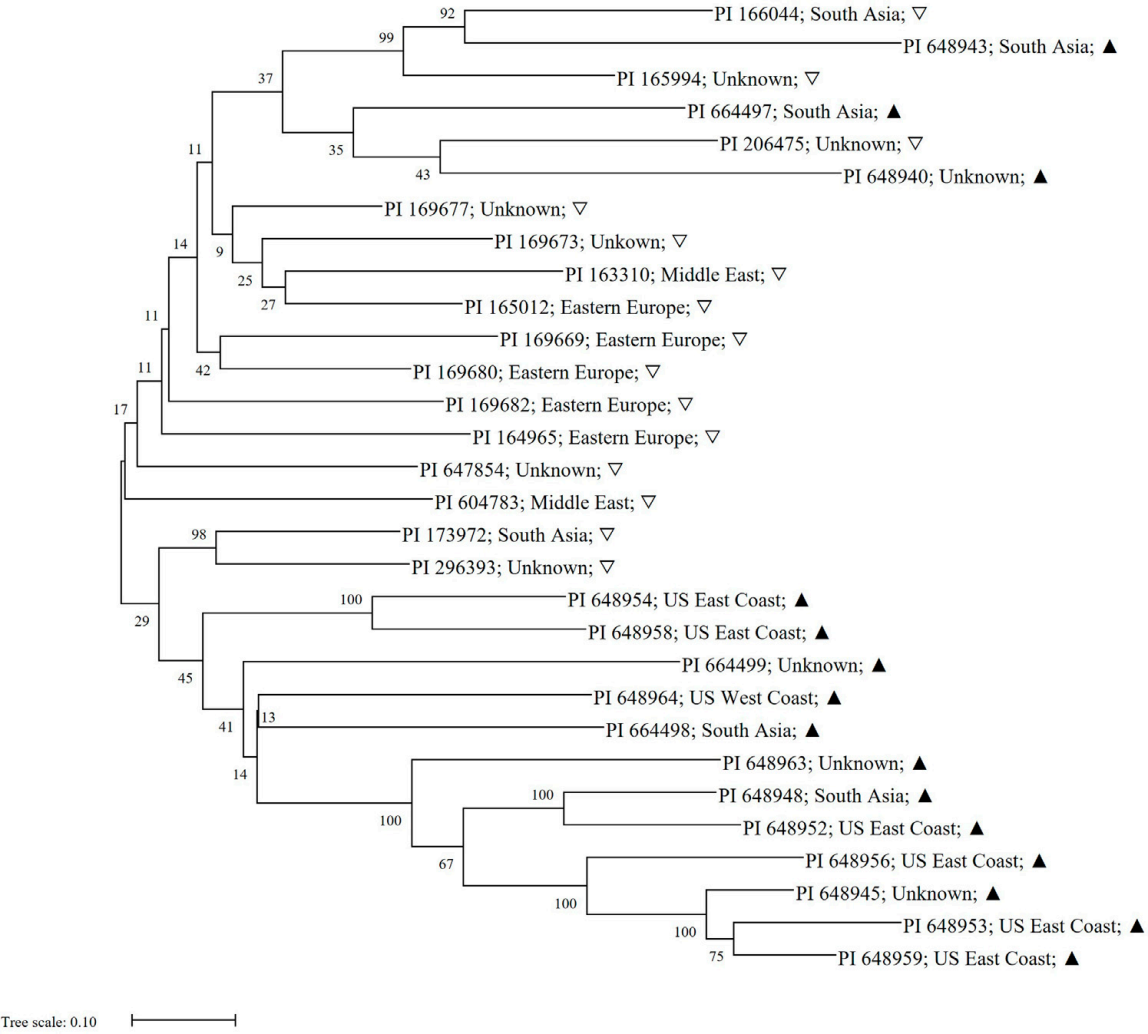
Ancestry relationships of spinach accessions with respect to 490 significant polymorphic sites from GWAS analysis. The ancestry history was inferred using the neighbor-joining statistical method and 500 bootstrap replications. Next to each branch is the corresponding accession identifier, origin and AsA content (low = ▽; high = ▲). Bootstraps are next to each split.

Family relatedness test was performed using the 15 highest and 15 lowest AsA content accessions and the 490 significant polymorphic variants. A phylogenetic tree was constructed from 30 accessions based on the neighbor-joining statistical method (Saitou and Nei, 1987) (Figure 10). Two main divergent clusters were found within the tree when sorting the top high and low AsA content accessions. The first clade consisted of a 13 low AsA content accessions and three high AsA content accessions. The second clade had a shared ancestry, with the remaining 14 accessions. Within this clade, apart from two accessions (PI 173972 & PI 296393), only high AsA content accessions are found. This suggests that a portion of SNPs specific to accessions with high AsA content are associated with this trait and upon validation, could be used as part of a molecular breeding selection strategy.

Most of the high AsA content accessions in the phylogenetic tree are of US origin, suggesting unintentional or intentional breeding selection for high AsA content has been performed. The high AsA content accessions PI 648953, PI 648959, and PI 648945, along with the low AsA content accessions present in the first hereditary branches, could be used as parents to create segregating populations for AsA content marker validation.

## Conclusion

Ascorbic acid (AsA) is an important antioxidant with multiple functions in plants and is an essential nutrient in the human diet. Therefore, efforts have been made in many crops to enhance AsA content. Here, we report that the variability of AsA content in spinach germplasm is high ranging from 0.02 to 2.12 µmol/g FW in the 352 accessions tested (106-fold change) indicating that existing diversity can be utilized for cultivar improvement. One strategy to increase selection efficiency is to use molecular breeding approaches after identifying markers associated with the trait. In this study, we implemented three genome-wide association models. Each method identified a collection of unique but also overlapping markers, suggesting that a combination of methods can help to narrow down associated polymorphisms in spinach. The GLM method identified 274 significant markers, followed by 204 markers found by the P3D, and 158 markers identified by the perMarker model (Supplementary Table S2, Figure 4). A total of 490 SNPs were identified by at least one of the three models, where 27 SNPs were commonly identified by all three models and could be used as primarily target for marker validation. Further work is needed to validate the usefulness of the identified markers for molecular breeding programs, but initial bioinformatic analysis suggests that the identified SNPs differentiate between accession clusters differing in AsA content. High AsA content accessions PI 648953, PI 648959, and PI 648945 can be used as parental lines for trait introgression and to create segregating populations for genetic analysis. Identification of significantly associated markers to AsA content in all spinach chromosomes it is an indicative of its complex inheritance, therefore molecular breeding approaches such as genomic selection could be used to improve AsA content gain in selection.

## Data Availability

The raw FASTQ files presented in the study are deposited in the NCBI - SRA (https://www.ncbi.nlm.nih.gov/sra) repository, accession number PRJNA779442. Raw phenotypic data is available in Supplementary Material.
